# Novel Biomarkers in Sinonasal Cancers: from Bench to Bedside

**DOI:** 10.1007/s11912-020-00947-2

**Published:** 2020-07-29

**Authors:** Matt Lechner, Jacklyn Liu, Valerie J. Lund

**Affiliations:** 1 UCL Cancer Institute, University College London, 72 Huntley Street, London, UK; 2 Royal London Hospital, Barts Health NHS Trust, London, UK; 3 Royal National Throat, Nose and Ear Hospital/Head and Neck Centre, University College London Hospitals NHS Foundation Trust, London, UK; 4 UCL Ear Institute, University College London, London, UK

**Keywords:** Biomarkers, Therapeutic targets, Targeted therapies, Sinonasal cancer, Olfactory neuroblastoma, Esthesioneuroblastoma, Sinonasal melanoma, Sinonasal undifferentiated carcinoma (SNUC), Sinonasal neuroendocrine carcinoma (SNEC), Sinonasal adenocarcinoma, Sinonasal squamous cell cancer, Metastasis

## Abstract

**Purpose of Review:**

Sinonasal cancers are a heterogenous group of rare cancers for which histopathological diagnosis can be very challenging and treatment options are limited for advanced disease in particular. Here, we review the candidacy of novel diagnostic and prognostic biomarkers, and therapeutic targets for sinonasal cancers.

**Recent Findings:**

Molecular multidimensional analyses of sinonasal cancers have been lagging behind other major cancers, but there are numerous publications describing the discovery of novel candidate biomarkers, e.g. the methylation classifier, originally developed for brain cancers, and gene expression panels for the prediction of response to induction chemotherapy in sinonasal undifferentiated carcinoma. The most promising biomarkers are summarized and discussed further with regard to their clinical applicability and future potential.

**Summary:**

Many of the described novel biomarkers for sinonasal cancers will eventually overcome the pitfalls associated with the frequently non-specific immunohistological tests. With comprehensive, multidimensional molecular testing of these tumours in collaborative consortia projects, our better understanding of the molecular mechanisms of sinonasal cancers and their carcinogenesis will determine the most useful diagnostic and prognostic biomarkers, allow stringent multi-institutional validation and guide trials on targeted therapies.

## Introduction

Sinonasal cancers are a heterogenous group of tumours arising in the sinonasal region, comprising the nasal cavity and sinuses. These are a very rare cancer type with an incidence of 0.83 per 100,000 individuals and make up less than 20% of head and neck cancers [1]. Recent analysis of the SEER database (1973–2015) demonstrated 5-, 10- and 20-year survival at 45.7%, 32.2% and 16.4%, respectively [2]. These cancers are more common in men and survival is negatively impacted by increasing age at diagnosis, AJCC TNM stage and grade [2].

Poor survival is largely due to the biology of the different cancer types, the frequent presentation at an advanced stage of disease and limited treatment options for advanced disease, especially when surgery and radiotherapy are not a curative option anymore. Typically, initial presentation includes unilateral nasal obstruction and serosanguinous nasal discharge/epistaxis [3]. The symptoms can be misconstrued as more common benign nasal processes, leading to an increased time between symptom presentation and diagnosis. Complete surgical resection with negative margins is the treatment of choice but this is not always possible and surgery can be challenging due to the complex sinonasal anatomy and tumour extension into neighbouring structures such as the orbit and/or brain. Studies have assessed the outcome of an endoscopic surgical approach compared with the classical open approach showing similar outcomes when like is compared with like, if not better for the former approach in the case of malignant sinonasal melanoma [4, 5]. It can be argued that an endoscopic approach results in reduced morbidity and fewer complications. Post-operative radiotherapy is also recommended for most subtypes in an effort to mitigate local recurrence; furthermore, the introduction of intensity-modulated radiotherapy has demonstrated improved tumour coverage while keeping radiation dose at a minimum. Multi-modality treatments including chemotherapy are also used depending on the tumour subtype as well as individual centre experience. Systemic therapy is typically reserved for patients who cannot undergo surgery, and often limited to palliation. However, studies investigating the efficacy of neoadjuvant chemotherapy suggest that there may be a benefit for some sinonasal cancer types, specifically in reducing tumour volume to make complete surgical resection more easily obtained [6, 7].

Sinonasal cancers are made up of an increasing number of histological subtypes, the most common of which is squamous cell carcinoma (SNSCC), followed by intestinal-type adenocarcinoma (ITAC), which are mainly found in the nasal cavity, and antroethmoid region [2] and other subtypes, such as olfactory neuroblastoma (ONB), sinonasal neuroendocrine carcinoma (SNEC), sinonasal undifferentiated carcinoma (SNUC) and sinonasal melanoma. Accurate diagnosis can be challenging requiring a range of differentiating diagnostic markers, especially for some subtypes. With the advent of technologies such as next-generation sequencing, it has become possible to increase our understanding of the genetic and epigenetic profiles of cancers in order to develop improved diagnostic tools as well as targeted therapies. In sinonasal cancer, a plethora of studies have emerged in the past decade which have sought to accomplish these goals. In particular, molecular studies have sought to improve the differential diagnosis of the various subtypes in order to better allocate treatment and improve outcome. However, due to the rarity of sinonasal cancer, and its subtypes, molecular studies are still limited, and research is lagging behind other major cancers. In the following section, we describe the progress for some of the subtypes (Table 1).

**Table 1:** Summary table of most promising recently published biomarkers and therapeutic targets

SNC subtype	Biomarker	(Type)	Reference
ONB	Methylation classifier (developed for brain cancers)	Diagnostic	Capper et al. (2018, Nature) Capper et al. (2018, Acta N)
Sinonasal melanoma	NRAS (G12 hot-spot mutation) and KIT	Therapeutic	Chraybi et al. (2013) Turri-Zanoni et al. (2013) Wroblewska et al. (2019)
SNUC	IDH2 mutation	Diagnostic	Jo et al. (2017); Dogan et al. (2017) Takahashi et al. (2019)
	24 gene panel for prediction of response to chemotherapy	Predictive	
Sinonasal NUT midline carcinoma	Translocation of the gene encoding nuclear protein in testis (*NUTM1*)	Diagnostic	Stelow and Bishop (2017)
SNEC	Insulinoma-associated protein 1 (INSM1)	Diagnostic	Rooper, Bishop and Westra (2018)
Sinonasal adenocarcinoma	KRAS	Therapeutic	Lopez et al. (2012) Szablewski et al. (2013) Garcia-Inclan et al. (2012)
SNSCC	EGFR	Therapeutic	Udager et al. (2015) Li and Zhu et al. (2017) Quan et al. (2019)
	TrkB	Prognostic	
	TIL	Prognostic/therapeutic	

## Novel Biomarkers in Olfactory Neuroblastoma

As with other sinonasal tumour subtypes, diagnosis of ONB is challenging and suffers from various pitfalls, especially in high-grade tumours. While these tumours are typically negative for cytokeratin staining, positivity has been reported in up to 30% of ONBs. Both Mandarano et al. and Holbrook et al. have demonstrated CK18 positivity in their tumour cohorts [8, 9]. As such, despite morphological and immunophenotypical consistency, improved diagnostic methods are much needed. A recent study conducted by Capper et al. (2018) showcased the reliability of methylation-based tumour profiling for more accurate diagnosis using a classifier system developed for brain tumours [10••, 11]. Furthermore, their study highlighted the likely prevalence of misdiagnosis in this malignancy and suggested that histology alone may frequently misdiagnose cases. The authors identified 4 subgroups within their ONB cohort, which was based on differential methylation status. The ‘core’ ONB group aligned most closely with classical ONB, with a trend toward lower grade alongside highly recurrent chromosomal losses in chromosomes 1, 2, 3, 4, 8, 9, 10 and 12. Importantly, the authors identified a subgroup with high DNA and CpG island methylation as well as *IDH1* and *IDH2* mutations, which is likely identical to sinonasal *IDH2* carcinoma, a distinct entity recently identified within the group of sinonasal undifferentiated carcinoma [12, 13]. In ONB, the most robust method of prognostic determination is through a comprehensive analysis involving Hyams grading [14]. This system has been shown to be an accurate prognostic tool: a recent meta-analysis demonstrated the utility of Hyams grading in predicting metastasis and overall survival where high-grade ONB was associated with significantly worse 5- and 10-year overall survival and increased neck and distant metastasis [15]. This supports the use of the grading system as a pre-operative marker with the ability to guide surgeons, closely checking for nodal and distant disease and considering a neck dissection. Moreover, recent molecular profiling of ONBs has revealed a plethora of further potential prognostic and predictive biomarkers, as well as therapeutic targets. Topcagic et al. demonstrated the potential role for such studies in predicting response to chemotherapy [16]. Here, their data showed aberrations in markers such as *ERCC1, TOPO1, TUBB3* and *MRP1*, which are known to reflect sensitivity to cisplatin, irinotecan, vincristine and combination therapy, respectively. In addition, the authors demonstrated aberrations in the targetable Wnt/β-catenin signalling pathway as well as cell cycle master-regulator *TP53*, which may confer sensitivity to WEE kinase inhibitors. In a study assessing the prevalence of clinically relevant genomic alterations, defined as those associated with therapeutics in either clinical trials or already approved for routine use, Gay et al. revealed that roughly half of their tumour cohort harboured such genomic alterations with particular frequency in the PI3K/mTOR signalling pathway, as well as CDK-dependent cell cycle regulation [17]. In this same study, the authors demonstrate the potential efficacy of targeted therapies such as everolimus, sunitinib and pazopanib, which they allocated based on the patient tumour’s molecular profile. Interestingly, Gallia et al. showed a high frequency of deletions in the *DMD* gene, which encodes for the structural protein dystrophin and is aberrated in various muscular dystrophies [18]. The authors point to previous studies, which demonstrated the tumour-suppressive role of *DMD*, highlighting the potential utility of this specific aberration as a therapeutic target. In line with the recent advancement in immunotherapies, Yu et al. showed 40% PD-L1 positivity in their tumour cohort with an associated increase in PD1^+^ and CD8^+^ lymphocyte infiltration in the tumour and stroma. This opens the door for studies assessing the efficacy of PD-1 and PD-L1-based therapies, such as nivolumab and pembrolizumab.

## Novel Biomarkers in Sinonasal Melanoma

Sinonasal melanoma is a highly aggressive, mucosal tumour type, characterized by early recurrence and may or may not respond to radiotherapy. Prognosis for these is less than 30% survival at 5 years. These tumours differ from their cutaneous counterparts specifically with a lack of *BRAF*V600E mutations and as such will likely be unresponsive to *BRAF*-targeting therapies. In two studies, this mutation was not detected in any case. However, recent studies have detected the aberration in 2 of 28 and 4 of 72 cases. Interestingly, mutations in *BRAF* (both at the V600 and D594 codons) approached significance in their correlation with overall and progression-free survival [19, 20]. The mutational status of *NRAS* and *KIT* have also been investigated with varying results. *NRAS* mutations have been reported in 4.8–22%; the lower percentage was likely due to combination of the study cohort with other head and neck mucosal melanomas. In comparison, *KIT* mutations have occurred in 0–22% of sinonasal melanomas [19–23]. While some studies have suggested that *NRAS* mutations largely occur at codons 12 and 13, in comparison with cutaneous melanomas which harbour mutations at codon 61, a study conducted by Wroblewska et al. consisting of 95 cases demonstrated a variety of mutations outside of the previously described hot-spots. As such, the difference between mucosal and cutaneous melanoma and the mutational landscape of *NRAS* in sinonasal melanoma is likely more complicated than previously thought. The prevalence of *KIT* mutations demonstrates the potential for KIT inhibitors as therapy; however, there appears to be geographical dependency as a cohort from Southern Italy displayed no *KIT* mutations while the larger, international case study mentioned above reported mutations in 22% of cases [20, 22].

Loss of *PTEN* and p16/INK4a may indicate activation of the PI3K/Akt/mTOR and RAS/MAPK pathways, which may in turn serve as potential therapeutic targets [23]. A more recent study has also detected mutations in the *TERT* promoter (3/28 cases) [19]. These mutations were first identified in melanoma and are known to create binding sites for Ets family transcription factors, which leads to upregulation of the telomerase enzyme and consequent evasion of senescence [24–27]. Due to the lack of potential biomarkers for this disease, Grunmuller et al. sought to identify a panel of targetable genes, which resulted in the generation of a biopanel including *KIT*, *TP53*, *MYC*, *HER2*, *EGFR*, *MET*, *VEGFR*, *BRAF*V600E and/or *MDM2* with loss of *ALK*, *FLI1* and *PDGFRa* [28]. This study did not assess the diagnostic, prognostic or predictive utility of these potential biomarkers; nevertheless, the evidence suggests that one or more of these genes and their associated pathways may serve as therapeutic targets.

Recent studies have investigated the use of various immunotherapies and targeted therapies in this disease context. Sayed et al. found no significant improvement in their patients who received adjuvant targeted or immunotherapy, including sorafenib, imatinib and ipilimumab [29]. However, as the authors note, this lack of improvement may actually be demonstrating some benefits as this treatment was generally given to those with end-stage, disseminated disease. Therefore, as the patients were not significantly worse than the comparator cohort, it may be that the use of such therapies might be beneficial. A recent systematic review and meta-analysis assessing the efficacy of endoscopic surgical resection versus open surgery also investigated the efficacy of ipilimumab in metastatic mucosal melanoma, as a whole, and demonstrated a 12.5% response rate, which improved to 23% in combination with the anti-PD1 therapy, nivolumab [30]. Thus, there may be a role for immunotherapies and targeted therapies in this subset of sinonasal cancers; however, the mechanisms of action are not well understood and currently there is no commonly used companion biomarker to assess candidacy.

## Novel Biomarkers in Sinonasal Undifferentiated Carcinoma

Sinonasal undifferentiated carcinoma (SNUC), first identified by Frierson in 1986, is generally regarded as a diagnosis of exclusion with complexities in the definition of its molecular, immunohistological and morphological characteristics. It is a highly aggressive tumour, which typically presents at an advanced stage with extensive disease locally, such as dural involvement and intracranial extension/invasion and sometimes cervical metastases. In addition to the frequently advocated combination of surgery and post-operative radiotherapy, it has been suggested that in some cases, these tumours are highly chemosensitive and respond to induction therapy [31]. In patients with complete response to chemotherapy, the best survival is observed in the group of patients who receive chemoradiotherapy. In a follow-up paper, Takahashi et al. identified a 24 gene expression panel that can distinguish between the two groups [32••]. As SNUC can appear histologically similar to other subtypes, such as large cell neuroendocrine carcinoma and high-grade olfactory neuroblastoma, misdiagnosis is not uncommon. Furthermore, even though the majority of tumours will stain negative for neuroendocrine markers, there is a proportion which stains positive, further blurring the line between SNUC and bonafide neuroendocrine carcinoma.

More recently, the identification of *IDH2* mutations in SNUCs has led to the emergence of a new subtype, which appears to be exclusive to SNUCs and has also been shown to distinguish this group from the core-olfactory neuroblastoma group by molecular profiling [10••, 12, 13]. Mutations at the R172 codon have been reported to occur in 55–82% of SNUC cases, prompting investigation into its utility as a diagnostic biomarker as well as the efficacy of IDH2 inhibitors in this cancer type. In relapsed or refractory acute myeloid leukaemia, the IDH2 inhibitors enasidenib and ivosidenib have recently obtained FDA approval, and clinical trials are ongoing [33–35]. Since the mutation almost always occurs at this position, Dogan et al. have determined the utility of the 11C8B1 monoclonal antibody as surrogate marker for this mutation [36]. In their study assessing its specificity amongst a cohort of tumours within the differential diagnosis for SNUC, they found that this antibody stained virtually exclusively in the SNUC subgroup. Consequently, it will likely be a useful diagnostic tool in the detection of *IDH2*-mutant undifferentiated carcinoma. Interestingly, a recent study conducted by Dogan et al. found that IDH2-mutant SNUCs were associated with global hypermethylation and upregulation of the repressive H3K27 epigenetic mark and were associated with improved DFS and reduced lung metastasis [37]. The authors suggest that DNA methylation-based classification of tumours may improve the accuracy of diagnosis, an important goal for this phenotypically heterogeneous group of tumours.

The SWI/SNF chromatin remodelling complex is made up of subunits, which have been shown to be aberrated in various cancers and may provide potential therapeutic targets [38, 39]. In poorly or undifferentiated sinonasal carcinoma, including SNUC, loss of *SMARCB1* and *SMARCA4* have been identified and are emerging as distinct entities in their own right. Sinonasal SMARCB1-deficient carcinomas were first identified independently by Bishop et al. and Agaimy et al. in 2014, characterized by deletions of the *SMARCB1* gene with variable staining for neuroendocrine markers and a mainly basaloid morphology, resembling SNUC or non-keratinizing SCC [40, 41].

In a recent cohort of 39 patients, 56% had died of disease at last follow-up (0–102 months), demonstrating the aggressive nature of these poorly or undifferentiated tumours [42]. Furthermore, a recent study determined worse prognosis for *SMARCB1*-deficient SNUC compared with the *SMARCB1*-retained SNUC cohort in OS and DFS with higher recurrence and mortality rates [43]. While this study is limited by a small sample size, the statistical significance achieved suggests an important prognostic role for *SMARCB1* status. Comparatively, *SMARCA4*-deficient SNUCs have also been identified and are mutually exclusive from their *SMARCB1* relatives while being morphologically identical. In the initial case, the authors determined *SMARCA4* loss, which is exclusive of *IDH2* mutations, as a potential oncogenic driver [44]. An additional 10 cases were assessed and underscored *SMARCA4*-deficient carcinoma as a genetically distinct entity [45]. Importantly, due to the heterogeneity of diagnosis for poorly and undifferentiated sinonasal carcinomas, misdiagnosis occurs often. In these studies, many of the tumours eventually identified as *SMARCA4* or *SMARCB1*-deficient were previously diagnosed as other entities, such as small and large cell neuroendocrine carcinoma. Thus, due to the aggressive nature of SMARC-deficient tumours, it is important to continue to refine the diagnostic parameters of these complicated tumours. Lastly, the identification of SWI/SNF complex proteins as key players in sinonasal cancer prompts intrigue in the potential efficacy of novel targeted therapeutics. In malignant rhabdoid tumours, which are driven by pathogenic loss of *SMARCB1*, various drugs targeting epigenetic regulation have been investigated [46]. In non-small cell lung cancer, evidence has shown that loss of *SMARCA4* leads to cyclin D1 deficiency. The authors, here, were able to demonstrate the synthetic lethality of *SMARCA4* loss with CDK4/6 inhibitors [47]. In addition, a recent study demonstrated the susceptibility of *SMARC4A*-deficient ovarian and lung cancer models to bromodomain inhibitors [48]. Whether this applies in sinonasal carcinoma warrants investigation.

In addition to the specific genomic aberrations discussed above, more comprehensive genomic and epigenomic profiling has been done to better understand the aetiology of disease in conjunction with improved diagnosis and potential prognosis. In one recent study, Takahashi et al. identified seven genes which are differentially expressed between SNUCs and squamous cell carcinoma, the most different of which is the gene *CLCA2* [49]. An immunohistochemical test is currently being developed to assess the diagnostic utility of this potential biomarker. Interestingly, in this study, the differentially expressed genes were involved in DNA repair, synthesis and replication as well as protein modifications and cell division. *CLCA2* encodes for a chloride transporter targeted by the p53 signalling pathway and is shown to have reduced expression in SNUCs compared with SCCs. Recent in vivo studies in nasopharyngeal carcinoma demonstrated inhibition of proliferation, migration, invasion and epithelial-mesenchymal transition through overexpression of *CLCA2* [50]. This is thought to occur through Fak/Erk signalling [51]. As such, the potential for the restoration of *CLCA2* activity in SNUCs as therapy is intriguing.

## Novel Biomarkers in Sinonasal NUT Midline Carcinoma

In the realm of poorly and undifferentiated carcinoma, further delineation of likely subtypes with distinct biological characteristics continues in an effort to improve diagnosis, prognosis and therapeutic targeting. Recent advances in molecular profiling have resulted in the emergence of subtypes, such as the sinonasal NUT carcinoma, which came from the identification of SNUCs with translocation of the gene encoding nuclear protein in testis (*NUTM1*). The 4th edition of the World Health Organization classification of head and neck tumours effectively separates NUT carcinoma from SNUC as its own tumour type, due to its aggressive disease course [52••].

## Novel Biomarkers in Sinonasal Neuroendocrine Carcinoma

Sinonasal neuroendocrine carcinoma (SNEC) is an aggressive epithelial, poorly differentiated tumour type, which typically present at an advanced stage and is associated with early distant metastases in roughly half of cases. Due to the extreme rarity of this disease, very few studies have been published concerning potential biomarkers for improved diagnosis, prognosis and treatment. The two main types of SNECs are small and large cell (SCNEC and LCNEC, respectively) and are associated with poor survival and high recurrence and metastasis rates. Due to their morphological similarity to SNUCs, staining of neuroendocrine markers is key in diagnosis and establishing the correct diagnosis remains challenging.

Like other sinonasal cancers, complete surgical resection is the mainstay of treatment; in some cases, chemoradiation is used with variable outcome. Interestingly, there may be a role for neoadjuvant chemotherapy and concurrent CRT, but this has only been shown in a single case report [53].

Few comprehensive molecular studies have been conducted to date, the majority of which look at multiple tumour types. Lopez and co-workers’ classification of subtype based on specific marker expression and copy number alterations demonstrated that SNECs typically stain positive for both cytokeratin and neuroendocrine markers, in contrast to SNUCs and ONBs and exhibit many copy number alterations with a high level of chromosomal instability [54]. In another study assessing the status of MiR-21 as a potential prognostic marker, the authors found that the marker was upregulated in their tumour cohort, which consisted of a small minority of SNECs [55]. An important limitation of these studies is that the authors did not separate SNEC into its small and large cell entities. Studies have demonstrated that these are indeed distinct subtypes and as such have different biological and clinical outcomes. Therefore, it is important that studies carefully differentiate between the two.

For neuroendocrine carcinoma specifically, a recent study investigated the status of insulinoma-associated protein 1 (*INSM1*) in a variety of head and neck neuroendocrine carcinomas [56]. Here, the authors found that all cases were positive for this biomarker so it may serve as a potential diagnostic biomarker for neuroendocrine carcinoma, improving on currently used markers which are not specific to SNECs alone.

## Novel Biomarkers in Sinonasal Adenocarcinoma

Sinonasal adenocarcinomas comprise intestinal-type and non-intestinal type tumours (ITAC and NITAC, respectively) with a combined 5-year overall survival of 54% [57]. Prognostic factors include age at diagnosis and tumour grade. Importantly, ITACs are associated with long-term occupational exposure to hard wood dust, which is thought to drive carcinogenesis. In addition, leather dust exposure has also been demonstrated to be an important risk factor [58–61]. Treatment recommendations involve complete surgical resection, when possible, using either the open or the endoscopic approach. Post-operative radiotherapy is also usually recommended [62]. Endoscopic surgery has been deemed safer and minimally invasive, with improved post-operative quality of life [63, 64].

The genomic and epigenomic profile of these tumours is not well understood; however, a recent study conducted by Lopez-Hernandes et al. demonstrated the utility of subgrouping ITACs into ‘clusters’ through copy number alteration assessment [65]. The authors identified 5 clusters with distinct prognoses, which were associated with subtype (i.e. papillary and mucinous), p53 status and intracranial invasion. A previous study assessing gene promoter methylation demonstrated a pattern, which differentiated SNSCC from ITAC [66]. Here, the authors found recurrent promoter methylation in ITAC of the genes *CDH13*, *ESR1*, *APC*, *TIMP3*, *CASP8*, *HIC1* and *RASSF1*. Interestingly, *TIMP3* methylation correlated with worse survival, which has been similarly demonstrated in gastric, colon, prostate and hepatic adenocarcinoma, indicating that epigenetic regulation of this gene may be an important driver in the carcinogenesis of this tumour type. In a different study assessing copy number alterations in ITAC, a pattern of recurrent gains and losses was noted with potential effects on the PI3K/Akt/mTOR pathway through *PTEN* loss, cell cycle regulation through aberrations of *TP53*, loss of *APC* which may affect Wnt signalling and other alterations involving p14 and p16, *DCC*, *DPC4* and *SMAD2* and *SMAD4* [67]. While the authors did not investigate correlations with clinicopathological characteristics nor outcome, this type of study brings to light potential targetable pathways for therapeutic development.

With regard to other potential biomarkers, Tomasetti et al. investigated the status of MiR-126 and found that it is reduced in ITACs compared with benign tumours, suggesting the potential for this MiRNA to act as a circulating biomarker for the detection of malignant transformation [68]. In vitro experiments demonstrated reduced cell growth and increased tumorigenic potential with the restoration of MiR-126. Thus, it may serve as a potential therapeutic pathway. *SATB2* has been recently identified as a potential diagnostic biomarker, capable of differentiating ITAC from NITAC with a high degree of specificity [69]. In this study, the authors demonstrate positive staining in 7 of 7 ITAC specimens and 0 of 66 in their NITAC cohort. Importantly, classical markers, such as CDX2 and CD20, are not absolutely specific for ITAC, with the potential for misdiagnosis likely with other tumour types such as SNUC.

Interestingly, a study conducted by Pirrone et al. demonstrated differential immunoreactivity of *OTX1* and *OTX2* between the intestinal and non-intestinal types where *OTX1* is only absent from ITACs while *OTX2* is absent from both [70]. The authors postulate that this difference may be due to the loss of a respiratory type epithelial phenotype, which may occur through loss of both genes. Two studies conducted by Andreasen et al. detected 3 cases of *ETV6* rearrangement in low-grade NITAC, two of which with *NTRK3* and one with *RET* which may serve as targets for therapy and diagnostic biomarkers in the future [71, 72].

Briefly, few recurrent mutations have been noted in sinonasal adenocarcinoma. The most frequently demonstrated is mutation of *KRAS*, which has been detected in 12–43% of cases, with a potential link to wood dust-associated carcinogenesis [67, 73–75]. In an effort to determine the utility of EGFR-based therapeutics, *BRAF* and *EGFR* status have also been investigated and have revealed that there are likely no genetic bases for such treatment [67, 73–75]. With regard to HER2 status, while previous studies have demonstrated some amplification, a recent report of 43 cases determined no cases with HER2 amplification [76]. However, evidence remains mixed and does not completely rule out these investigative pathways as improved technologies enable better assessment of the genetic and epigenetic landscape of these tumours.

## Novel Biomarkers in Sinonasal Squamous Cell Cancer

Squamous cell carcinomas of the sinonasal tract are the most common tumour type to arise in sinonasal cancer. In head and neck squamous cell carcinoma, *EGFR* has been found to be overexpressed in a significant proportion of cancers [77, 78]. As a consequence, studies have sought to determine the expression pattern of the gene in the sinonasal subtype. In addition, since a proportion of SCCs arise from the malignant transformation of inverted papilloma (ISP), there has been research into the identification of putative markers for the carcinogenesis of these benign and common tumours. In a study conducted by Udager et al., the authors found that there was a statistically significant difference in *EGFR* expression between ISPs and ISP-associated SNSCC [79]. It is thought that these mutations may lead to constitutive activation and serve as a therapeutic target. Interestingly, the authors found that the irreversible *EGFR* inhibitor, neratinib, strongly inhibited *EGFR* signalling and its downstream molecules Mek and Akt in an SNSCC cell line. In addition, the difference in prevalence of *EGFR* mutations in these two tumour cohorts suggests that such mutations are early events in ISP pathogenesis. Furthermore, the authors suggest that ISP-associated SNSCC is a biologically distinct entity from other SNSCCs. In a comprehensive genomic profile of ISP-associated SNSCC, Yasuwaka et al. found that there were significantly more mutations in SNSCCs compared with ISP [80]. In addition, *KRAS* and *APC* were found to be more frequently altered in dysplasia and carcinoma. Here, *KRAS* was predictive for these with 85% sensitivity and 90% specificity. Treatment of sinonasal SCC with a combination of radiotherapy and cetuximab has been reported [81].

*TP53* is the most widely aberrated gene in cancer; while its status in sinonasal cancer is more controversial, there appears to be a role for the gene in the carcinogenesis of SNSCC. In two studies conducted in 2017, both found aberrant p53 expression by IHC. Vital et al. demonstrated an aberrant staining pattern in 60% of their cohort of SCC of the nasal vestibule, which was associated with worse DFS [82]. Wang et al. found higher p53 expression in SNSCC compared with benign papillomas and normal mucosa, which was associated with poor to moderate differentiation [83].

With regard to a potential prognostic marker, Li and Zhu assessed 27 cases of SNSCC by IHC and found overexpression of TrkB in all cases, which was related to moderate to poor differentiation, high clinical stage, presence of local recurrence and shorter OS and DFS [84]. In their analysis, TrkB was an independent prognostic factor for OS and DFS. In a more recent study, Munoz-Codero et al. determined pS6 as a potential negative prognostic indicator where overexpression was associated with more advanced stage and grade as well as worse OS and DFS [85]. This was further observed alongside *PTEN* loss and overexpression of Akt and mTOR, suggesting activation of the PI3K/Akt/mTOR pathway in these tumours.

Lastly, a recent study assessing PD-L1 status in SNSCC demonstrated 30% positivity, which was correlated with poor differentiation and high levels of tumour-infiltrating lymphocytes (TILs) [86]. While PD-L1 expression was not associated with survival, CD8^+^ TILs were prognostic for OS and DFS. As such, the utility of PD-L1 targeting and other immunotherapies should continue to be investigated.

## Discussion

These results offer a starting point for the development of novel biomarkers for sinonasal cancers, which do not suffer from the pitfalls associated with immunohistological tests which are frequently less specific. However, the correlation of an observation or a measurement to a phenotype which is described in most studies, with the aim of developing a biomarker, is frequently not sufficient. As an observational method, it lacks the strengths of an empirical approach, such as in a designed experiment. Kern stated that less than 1% of published cancer biomarkers enter clinical practice and summarized various reasons why this could be the case [87]. These include a lack of clinical utility, a technically inadequate assay, inappropriate statistical and inferential methods, normal variations dominating the observations and deficiencies in either the studied populations or the investigator system. Hence, the described observations with regard to genetic and epigenetic changes in sinonasal cancers may be very difficult to utilize as a biomarker. Considerable effort is needed to overcome the various hurdles as a consequence of which 99% of all published cancer biomarkers eventually fail to enter clinical practice to become diagnostic and prognostic candidate markers for sinonasal cancer. Further in vitro and in vivo experiments will help to contribute to the understanding of genetic and epigenetic changes at oncogenic loci associated with the progression of sinonasal cancer. These future efforts linked with prospective clinical studies will potentially advance the further developments of the most promising markers and identify putative therapeutic targets. The different biology and clinical behaviour of the various subtypes of sinonasal cancer and its overall rarity make undertaking large, multi-centre studies necessary. Efforts to standardize diagnosis across institutions and consider improved diagnostic methods should be paramount to improve on the validity, accuracy and consistency of diagnosis, which is the first step in the establishment of robust future studies. The comprehensive, multidimensional molecular testing of these tumours, leading to a better understanding of the molecular mechanisms of disease, will naturally identify the most useful diagnostic and prognostic biomarkers and at the same time identify new therapeutic targets. The candidate biomarkers, highlighted throughout this review, should be regarded as the starting point which directs further testing and stringent validation.

## Summary

Although many of above candidate markers appear to be very promising, they will need to undergo stringent testing to identify the most specific, clinically relevant markers which will be closely linked to the underlying biology and carcinogenic processes, helping to build a comprehensive model of carcinogenesis for each sinonasal cancer subtype. This will finally boost the translation of personalized cancer medicine into the clinical management of sinonasal cancers, a group of cancers which has only now been subjected to the multidimensional molecular analyses which have already been completed for many other common cancers. Large consortia projects are now aiming to develop biomarkers based on genomic and epigenomic data, such as the 100.000 Genomes Project (https://www.genomicsengland.co.uk/about-gecip/gecip-domains/). This has recently completed the whole-genome sequencing of more than 100.000 samples, including sinonasal cancers, which will significantly boost this trend. From here onwards, the sharing of data by international collaborations will overcome the hurdles inherent to such rare diseases, such as sinonasal cancers. This will eventually lead to the development of clinically relevant biomarkers, which is of the utmost importance in order to diagnose these tumours accurately, inform about prognosis and identify new therapeutic targets for advanced diseases, progress which will surely lead to improved survival for this challenging group of cancers.
